# Complete genome sequences and characteristics of mycobacteriophages Diminimus, Dulcita, Glaske16, and Koreni

**DOI:** 10.1128/MRA.01010-23

**Published:** 2023-12-08

**Authors:** Faith W. Baliraine, Kaitlyn E. Mathews, Emma G. Livingston, Clarissa A. Martinez, Olivia L. Donnelly, Taryn M. Pledger, Tadeen Feroz, Zoe J. Harbison, Sarah G. Schlimme, Camila Andrade, Keren N. Salazar, Elise C. Berryhill, Madelyn M. DeLosSantos, Hannah L. Foree, Wanjiru Gicheru, Adrienne M. Jett, Sofia N. Mendez, Toluwalope M. Odebiyi, Jacob I. Pitman, Michael J. Tan, Josh D. McLoud, Frederick N. Baliraine

**Affiliations:** 1 Department of Biology and Kinesiology, LeTourneau University, Longview, Texas, USA; 2 Department of Electrical, Computer, and Biomedical Engineering, LeTourneau University, Longview, Texas, USA; Department of Biology, Queens College, Queens, New York, USA

**Keywords:** mycobacteriophages, bacteriophages, Diminimus, Dulcita, Glaske16, Koreni

## Abstract

Complete genome sequences of four novel mycobacteriophages, Diminimus, Dulcita, Glaske16, and Koreni, isolated from soil are presented. All these bacteriophages belong to subcluster M1, except Koreni that belongs to subcluster A4. Moreover, all have siphovirus morphologies, with genome sizes ranging from 51,055 to 81,156 bp.

## ANNOUNCEMENT

Bacteriophages are obligate intracellular parasitic viruses of bacteria (1, 2). These viruses play crucial roles in the global ecosystem and impact not only bacterial physiology, diversity, abundance, and virulence but also human, animal, and plant health (3, 4). On the practical side, bacteriophages have multiple applications, including disease diagnosis, phage therapy, food safety, disinfection, correcting dysbiosis, pest control, biosensing, and bioremediation (4 – 9). Here, we report on four novel lysogenic bacteriophages.

All bacteriophages were isolated from soil samples collected around LeTourneau University in Longview, Texas, in August 2022 (Table 1), using standard protocols (10). Briefly, soil samples were mixed with Middlebrook 7H9 broth prior to spinning (2,000 × *g* at 4°C) and supernatant filtration (0.22 µm pore size). Filtrates were inoculated with *Mycobacterium smegmatis* mc^2^155 cells and incubated at 37°C for 4 days with shaking at 210 rpm, then filtered again. The samples were then plated with *M. smegmatis* in 7H9 top agar and purified through three 48-h rounds of plating at 37°C. Plaque morphologies were clear (Table 1). Negative-stain transmission electron microscopy showed the four bacteriophages to have a siphovirus morphotype with isometric capsids (diameter, ~60.75 to 68.21 nm) and flexible tails (length, ~131.60 to 333.00 nm; Table 1), measured using ImageJ (11 – 13).

**TABLE 1:** Properties of four mycobacteriophages isolated from soil samples collected on August 23, 2022 in Longview, Texas, USA

Data for mycobacterium phage^*a*^				
Characteristic	Diminimus	Dulcita	Glaske16	Koreni
Soil sampling location GPS coordinates	32.46474 N , 94.7272 W	32.464444 N , 94.7275 W	32.465 N , 94.727778 W	32.463333 N , 94.726389 W
Lysate titer (PFU/mL)	1.8 × 1,010	2.0 × 1,010	4.9 × 109	1.4 × 1,010
Plaque morphology after 48 h at 37°C	Clear with defined edges	Clear with defined edges	Clear with defined edges	Clear with defined edges
Avg plaque diameter (mm [n-value])	0.8 (15)	1.0 (10)	1.7 (3)	1.0 (13)
Approx. shotgun coverage (X)	338	479	360	1,490
Genome size (bp)	80,037	80,038	81,156	51,055
GC content (%)	61.6	61.6	61.6	63.9
Overhang sequence	ACCTCCTGCAA	ACCTCCTGCAA	ACCTCCTGCAA	CGGCCGGTAA
Overhang length (bases)	11	11	11	10
Cluster	M	M	M	A
Subcluster	M1	M1	M1	A4
GenBank accession no.	OR521083	OR553916	OR553909	OR553901
SRA accession no.	SRX19690831	SRX19690832	SRX19690837	SRX19690842
Total no. of reads	185,644	258,101	201,637	528,548
No. of predicted genes	137	137	140	90
No. of predicted tRNAs	19	19	18	0
tRNA type(s)	Arg, Asn, Asp, Cys, Gln, Glu, Gly, His, Leu, Lys, Met, Phe, Pro, Ser, Thr, Trp, Tyr, Val	Arg, Asn, Asp, Cys, Gln, Glu, Gly, His, Leu, Lys, Met, Phe, Pro, Ser, Thr, Trp, Tyr, Val	Trp, Asn, Gln, Tyr, Pro, Ser, Phe, Met, Arg, His, Leu, Lys, Gly, Val, Thr, Asp, Glu	–
No. of genes with predicted functions	51	52	54	48
% of genes with predicted functions	37.2	38	38.6	53.3
Key predicted lysogenic life cycle genes	Serine integrase	Serine integrase	Serine integrase	Serine integrase, immunity repressor
No. of orphams	0	0	1	0
Avg capsid size (nm [n-value])	61.53 (10)	60.75 (10)	68.21 (3)	61.56 (10)
Avg tail length (nm [n-value])	323.51 (10)	333.00 (11)	328.30 (3)	131.60 (10)
Isolated by	Faith W Baliraine, Taryn M Pledger, Camila Andrade, Chloe I Wade	Krista L Anderson,Olivia L Donnelly,Hannah L Foree, Kaitlyn E Mathews	Sofia N Mendez, Tadeen Feroz, Amya R Orn, Jerron Hudson	Krista L Anderson,Olivia L Donnelly,Hannah L Foree, Kaitlyn E Mathews

^*a*^ All bacteriophages were isolated using the enriched isolation method [reference (10)] and purified through three sequential (37°C, 48 h) rounds of plating with *Mycobacterium smegmatis* mc^2^155 cells in Middlebrook 7H9 top agar. Genome sequencing was accomplished using the Illumina Shotgun sequencing method with 150-base single-end reads using the NEB Ultra II Library sequencing kit (v3 reagents). All had 3′ single-stranded overhang genome ends. Genomic termini were identified through buildups of read start positions and variations in genome-wide coverage and manually verified using Consed version 29 [references (14) and (15) ]. All bacteriophages had a siphovirus morphotype and were predicted to be lysogenic based on the presence of predicted lysogeny-related genes.

Genomic DNA was extracted from lysates of titers ranging from 4.9 × 10^9^ to 2 × 10^10^ PFU/mL (Table 1) using the Promega Wizard DNA cleanup kit. The NEB Ultra II Library kit was used to prepare the samples for sequencing using Illumina MiSeq (v3 reagents; 150-base single-end reads). Untrimmed reads assembly and verification was performed using Newbler v2.9 (16) and Consed v29 (14, 15). Genome sizes ranged from 51,055 bp (phage Glaske16) to 81,156 bp (phage Koreni) (Table 1). All had 3′ single-stranded overhangs (10–11 bp long) and an average GC content of 62.2% (range: 61.6%–63.9%). This was slightly lower than that of our previous isolates from the same general location and of the isolation host *M. smegmatis* mc^2^155 (67.4% GC) (17, 18). Using the gene content similarity (GCS) tool in PhagesDB (19, 20), mycobacteriophages Diminimus, Dulcita, and Glaske16 were assigned to subcluster M1, while Koreni was assigned to subcluster A4 (Table 1) based on ≥35% GCS to other phages in the database.

The genomes were annotated using DNAMaster v5.23.6 (21), Starterator (22), Phamerator (23), BLASTp in NCBI GenBank and PhagesDB (24, 25), GenMark v2.5p (26), HHpred PDB_mmCIF70_17_Apr, Pfam-A_v35, UniProt-SwissProt-viral70_3_Nov_2021 and NCBI_Conserved_Domains_v3.19 databases (27, 28), Glimmer v3.02 (29), DeepTMHMM v. 1.0.24 (30), tRNAscan-SE v2.0 (31, 32), and ARAGORN v1.2.41 (33). Default program settings were utilized in all cases (34). On average, 126 putative protein-coding genes (range: 90–140) and 14 tRNAs (range: 0–19) were predicted (Table 1). Functions were predictable only for 37%–53% of the putative genes across the bacteriophages (Table 1). All bacteriophages had at least one lysogenic lifecycle-associated gene. Diminimus, Dulcita, and Glaske16 encoded serine integrase, while Koreni encoded both serine integrase and an immunity repressor. None had an identifiable gene encoding the excise (Table 1).

## Data Availability

Raw reads of all four reported mycobacteriophages are available in the Sequence Read Archive (SRA) database, and their complete genome sequences are available in GenBank database. Their SRA and GenBank accession numbers, together with their respective Uniform Resource Locators, are provided in Table 1. Plaque and TEM images are available in the Actinobacteriophage database and can be accessed by typing the phage name in the database search box. High titer lysates of the phages are archived at the University of Pittsburgh Bacteriophage Institute.

## References

[1] Jacobs-Sera D , Marinelli LJ , Bowman C , Broussard GW , Guerrero Bustamante C , Boyle MM , Petrova ZO , Dedrick RM , Pope WH , Modlin RL , Hendrix RW , Hatfull GF , Science Education Alliance Phage Hunters Advancing Genomics and Evolutionary Science (SEA-PHAGES) program . 2012. On the nature of mycobacteriophage diversity and host preference. Virology 434:187–201. doi:10.1016/j.virol.2012.09.026 23084079 PMC3518647

[2] Boeckaerts D , Stock M , Criel B , Gerstmans H , De Baets B , Briers Y . 2021. Predicting bacteriophage hosts based on sequences of annotated receptor-binding proteins. Sci Rep 11:1467. doi:10.1038/s41598-021-81063-4 33446856 PMC7809048

[3] Naureen Z , Dautaj A , Anpilogov K , Camilleri G , Dhuli K , Tanzi B , Maltese PE , Cristofoli F , De Antoni L , Beccari T , Dundar M , Bertelli M . 2020. Bacteriophages presence in nature and their role in the natural selection of bacterial populations. Acta Biomed 91:e2020024. doi:10.23750/abm.v91i13-S.10819 PMC8023132 33170167

[4] García-Cruz JC , Huelgas-Méndez D , Jiménez-Zúñiga JS , Rebollar-Juárez X , Hernández-Garnica M , Fernández-Presas AM , Husain FM , Alenazy R , Alqasmi M , Albalawi T , Alam P , García-Contreras R . 2023. Myriad applications of bacteriophages beyond phage therapy. PeerJ 11:e15272. doi:10.7717/peerj.15272 37101788 PMC10124553

[5] Jones HJ , Shield CG , Swift BMC . 2020. The application of bacteriophage diagnostics for bacterial pathogens in the agricultural supply chain: from farm-to-fork. Phage 1:176–188. doi:10.1089/phage.2020.0042 36147287 PMC9041468

[6] Dedrick RM , Freeman KG , Nguyen JA , Bahadirli-Talbott A , Cardin ME , Cristinziano M , Smith BE , Jeong S , Ignatius EH , Lin CT , Cohen KA , Hatfull GF . 2022. Phage therapy of mycobacterium 113 infections: compassionate-use of phages in twenty patients with drug-resistant 114 mycobacterial disease. Clin Infect Dis 76. doi:10.1093/cid/ciac453 PMC9825826 35676823

[7] Hatfull GF , Dedrick RM , Schooley RT . 2022. Phage therapy for antibiotic-resistant bacterial infections. Annu Rev Med 73:197–211. doi:10.1146/annurev-med-080219-122208 34428079

[8] Dedrick RM , Guerrero-Bustamante CA , Garlena RA , Russell DA , Ford K , Harris K , Gilmour KC , Soothill J , Jacobs-Sera D , Schooley RT , Hatfull GF , Spencer H . 2019. Engineered bacteriophages for treatment of a patient with a disseminated drug-resistant Mycobacterium abscessus. Nat Med 25:730–733. doi:10.1038/s41591-019-0437-z 31068712 PMC6557439

[9] Elois MA , Silva R da , Pilati GVT , Rodríguez-Lázaro D , Fongaro G . 2023. Bacteriophages as biotechnological tools. Viruses 15:349. doi:10.3390/v15020349 36851563 PMC9963553

[10] Poxleitner M , Pope W , Jacobs-Sera D , Sivanathan V , Graham H . 2018. Phage discovery guide. Howard Hughes Med Institute, Chevy Chase, MD.

[11] Schneider CA , Rasband WS , Eliceiri KW . 2012. NIH image to imagej: 25 years of image analysis. Nat Methods 9:671–675. doi:10.1038/nmeth.2089 22930834 PMC5554542

[12] Schindelin J , Arganda-Carreras I , Frise E , Kaynig V , Longair M , Pietzsch T , Preibisch S , Rueden C , Saalfeld S , Schmid B , Tinevez J-Y , White DJ , Hartenstein V , Eliceiri K , Tomancak P , Cardona A . 2012. Fiji: an open-source platform for biological-image analysis. Nat Methods 9:676–682. doi:10.1038/nmeth.2019 22743772 PMC3855844

[13] Rueden CT , Schindelin J , Hiner MC , DeZonia BE , Walter AE , Arena ET , Eliceiri KW . 2017. Imagej2: imagej for the next generation of scientific image data. BMC Bioinform 18:529. doi:10.1186/s12859-017-1934-z PMC5708080 29187165

[14] Gordon D , Green P . 2013. Consed: a graphical editor for next-generation sequencing. Bioinformatics 29:2936–2937. doi:10.1093/bioinformatics/btt515 23995391 PMC3810858

[15] Russell DA . 2018. Sequencing, assembling, and finishing complete bacteriophage egnomes. Methods Mol Biol 1681:109–125. doi:10.1007/978-1-4939-7343-9_9 29134591

[16] Margulies M , Egholm M , Altman WE , Attiya S , Bader JS , Bemben LA , Berka J , Braverman MS , Chen Y-J , Chen Z , Dewell SB , Du L , Fierro JM , Gomes XV , Godwin BC , He W , Helgesen S , Ho CH , Irzyk GP , Jando SC , et al. . 2005. Genome sequencing in microfabricated high-density picolitre reactors. Nature 437:376–380. doi:10.1038/nature03959 16056220 PMC1464427

[17] Weiss SM , Happy KK , Baliraine FW , Beach AK , Brobston SM , Martinez CP , Menard KJ , Orton SM , Salazar AL , Frederick GD , Baliraine FN , Stedman KM . 2023. Complete genome sequences and characteristics of seven novel mycobacteriophages isolated in East Texas. Microbiol Resour Announc 12. doi:10.1128/mra.00335-23 PMC10353418 37272813

[18] Mohan A , Padiadpu J , Baloni P , Chandra N . 2015. Complete genome sequences of a Mycobacterium smegmatis laboratory strain (MC2 155) and isoniazid-resistant (4XR1/R2) mutant strains. Genome Announc 3:e01520-14. doi:10.1128/genomeA.01520-14 25657281 PMC4319614

[19] Russell DA , Hatfull GF . 2017. PhagesDB: the actinobacteriophage database. Bioinformatics 33:784–786. doi:10.1093/bioinformatics/btw711 28365761 PMC5860397

[20] Pope WH , Mavrich TN , Garlena RA , Guerrero-Bustamante CA , Jacobs-Sera D , Montgomery MT , Russell DA , Warner MH , Hatfull GF . 2017. Bacteriophages of gordonia spp. mBio 8. doi:10.1128/mBio.01069-17 PMC5559632 28811342

[21] Retchless AC , Lawrence JG . 2007. Temporal fragmentation of speciation in bacteria. Science 317:1093–1096. doi:10.1126/science.1144876 17717188

[22] Shaffer C . 2016. Starterator. Available from: http://phages.wustl.edu/starterator

[23] Cresawn SG , Bogel M , Day N , Jacobs-Sera D , Hendrix RW , Hatfull GF . 2011. Phamerator: a bioinformatic tool for comparative bacteriophage genomics. BMC Bioinformatics 12:395. doi:10.1186/1471-2105-12-395 21991981 PMC3233612

[24] Altschul SF , Gish W , Miller W , Myers EW , Lipman DJ . 1990. Basic local alignment search tool. J Mol Biol 215:403–410. doi:10.1016/S0022-2836(05)80360-2 2231712

[25] Ismail HD . 2022. Bioinform:a practical guide to NCBI databases and sequence alignments. CRC Press.

[26] Borodovsky M , McIninch J . 1993. Recognition of genes in DNA sequence with ambiguities. Biosystems 30:161–171. doi:10.1016/0303-2647(93)90068-n 8374073

[27] Zimmermann L , Stephens A , Nam S-Z , Rau D , Kübler J , Lozajic M , Gabler F , Söding J , Lupas AN , Alva V . 2018. A completely reimplemented MPI bioinformatics toolkit with a new HHpred server at its core. J Mol Biol 430:2237–2243. doi:10.1016/j.jmb.2017.12.007 29258817

[28] Gabler F , Nam S-Z , Till S , Mirdita M , Steinegger M , Söding J , Lupas AN , Alva V . 2020. Protein sequence analysis using the MPI bioinformatics toolkit. Curr Protoc Bioinform 72:e108. doi:10.1002/cpbi.108 33315308

[29] Delcher AL , Harmon D , Kasif S , White O , Salzberg SL . 1999. Improved microbial gene identification with GLIMMER. Nucleic Acids Res 27:4636–4641. doi:10.1093/nar/27.23.4636 10556321 PMC148753

[30] Hallgren J , Tsirigos KD , Pedersen MD , Almagro Armenteros JJ , Marcatili P , Nielsen H , Krogh A , Winther O . 2022. DeepTMHMM predicts alpha and beta transmembrane proteins using deep neural networks. bioRxiv. doi:10.1101/2022.04.08.487609

[31] Lowe TM , Chan PP . 2016. tRNAscan-SE on-line: integrating search and context for analysis of transfer RNA genes. Nucleic Acids Res 44:W54–7. doi:10.1093/nar/gkw413 27174935 PMC4987944

[32] Chan PP , Lin BY , Mak AJ , Lowe TM . 2021. tRNAscan-SE 2.0: improved detection and functional classification of transfer RNA genes. Nucleic Acids Res 49:9077–9096. doi:10.1093/nar/gkab688 34417604 PMC8450103

[33] Laslett D , Canback B . 2004. ARAGORN, a program to detect tRNA genes and tmRNA genes in nucleotide sequences. Nucleic Acids Res 32:11–16. doi:10.1093/nar/gkh152 14704338 PMC373265

[34] Pope W , Jacobs-Sera D , Russell D , Cresawn S , Hatfull G . 2017. SEA-PHAGES Bioinformatics Guide. Univ Pittsburgh, Sci Educ Alliance-Phage Hunters Adv Genomics Evol Sci. Available from: https://seaphagesbioinformatics.helpdocsonline.com/home. Retrieved 20 May 2022.

